# Cellular viability effects of fatty acid amide hydrolase inhibition on cerebellar neurons

**DOI:** 10.1186/1755-7682-4-28

**Published:** 2011-08-19

**Authors:** Kathia Lueneberg, Guadalupe Domínguez, Oscar Arias-Carrión, Marcela Palomero-Rivero, Diana Millán-Aldaco, Julio Morán, René Drucker-Colín, Eric Murillo-Rodríguez

**Affiliations:** 1 Instituto de Fisiología Celular, División de Neurociencias Universidad Nacional Autónoma de México México DF, México; 2 Department of Neurology, Philipps University, D-35033 Marburg, Germany; 3 Laboratorio de Neurociencias Moleculares e Integrativas Escuela de Medicina, División Ciencias de la Salud Universidad Anáhuac Mayab Mérida, Yucatán. México

## Abstract

The endocannabinoid anandamide (ANA) participates in the control of cell death inducing the formation of apoptotic bodies and DNA fragmentation. The aim of this study was to evaluate whether the ANA degrading enzyme, the fatty acid amide hydrolase (FAAH), would induce cellular death. Experiments were performed in cerebellar granule neurons cultured with the FAAH inhibitor, URB597 (25, 50 or 100 nM) as well as endogenous lipids such as oleoylethanolamide (OEA) or palmitoylethanolamide (PEA) and cellular viability was determined by MTT test. Neurons cultured with URB597 (25, 50 or 100 nM) displayed a decrease in cellular viability. In addition, if cultured with OEA (25 nM) or PEA (100 nM), cellular death was found. These results further suggest that URB597, OEA or PEA promote cellular death.

## Introduction

Endogenous lipids have been the focus of interest since they display some biological functions. Among these molecules are oleoylethanolamide (OEA), palmitoylethanolamide (PEA) [1-3] as well as the endogenous agonist for cannabinoid receptors, arachidonoylethanolamine, also named anandamide (ANA) [4].

OEA is a naturally occurring fatty acid compound that modulates several neurobiological functions including satiety [3,5,6], displays diurnal fluctuations in several brain areas [7], and it has been related with fat ingestion [8]. On the other hand, PEA acts as an antinociceptive molecule [1,9] and displays anti-inflammatory properties [10].

The hydrolysis of ANA, OEA and PEA is catalyzed by an intracellular enzyme defined as fatty acid amide hydrolase (FAAH), for a comprehensive review see [11,12]. The activity of FAAH has been studied using highly selective inhibitors [13,14], including URB597 [3,5,6,15-17].

Pharmacologically ANA mimics many of the effects caused by Δ^9^- tetrahydrocannabinol, the primary psychoactive molecule of marijuana [18] on diverse behaviors such as memory disruption, hypolocomotion, hyperphagia, and sleep, for a comprehensive review see [19]. Although it has been reported that ANA induces cellular death [20-22], there is no solid evidence about the neurobiological role in cellular viability of URB597 as well as OEA or PEA. Thus, on the basis of these previous studies, we investigated whether these compounds would promote cellular death.

## Materials and methods

### Animals

Experiments were performed following the guidelines on the Ethical Use of Animals from the Mexican Institutes of Health Research (DOF. NOM-062-Z00-1999) as well as the National Institutes of Health Guide for the Care and Use of Laboratory Animals (NIH publication No. 80-23, revised 1996) and protocol was approved by the Committee on the Ethics of Animal Experiments of our Institutions. All efforts were made to minimize animal stress and suffering. C57B16/J mice (7-10 days old) of either gender were housed at constant temperature (21 ± 1°C) under controlled light-dark cycle (lights on: 07:00-19:00 h). Food and water were provided ad libitum.

### Compounds

Fetal calf serum and penicillin/streptomycin were obtained from GIBCO (Grand Island, NY, USA). Poly-L-lysine hydrobromide (molecular weight > 130,000), trypsin, DNAse, MTT (3-(4, 4dimethylthiazol-2-yl)-2,5-diphenyletrazolium bromide), cytosine β-D-arabino-furosamide were obtained from Sigma (St. Louis, Mo. USA). URB597, OEA, and PEA were kindly provided by Professor Daniele Piomelli (University of California, Irvine. USA). All drugs were dissolved in vehicle (polyethylglycol (PEG)/saline; 5:95 v/v). The doses (10, 25, 50 or 100 nM of each compound) were chosen from pilot experiments and they were administered randomly to the cultures.

### Cellular culture

Cerebellar granule neurons were obtained as previously described [23,24]. Briefly, animals were sacrificed by decapitation during the lights-on period (10:00 h) and the brain was rapidly removed and placed into a plastic matrix immersed in ice-cold with artificial cerebrospinal fluid. The cerebellum was collected (time collection < 5 min) and dissociated cell suspensions of cerebella were plated at a density of 265,000 cells/cm^2 ^in plastic dishes coated previously with poly-L-lysine (5 μg/mL) or in plastic dishes with coverslips using poly-L-lysine 25 μM. Culture medium contained basal Eagle's medium supplemented with 10% (v/v) heat inactivated fetal calf serum, 2 mM glutamine, 25 mM KCl (K25), D-(+)-Glucose (7.5 mM), 50 μg/mL streptomycin, and 50 U/mL penicillin. Culture dishes were incubated at 37°C in a humidified 5% CO_2_/95% air atmosphere, and cytosine arabinoside (10 μM). Control group consisted in cells incubated only with culture media whereas vehicle group was the culture with free-serum conditions and the respective solvent (vehicle). Separately, cells were treated with URB597, OEA or PEA (10, 25, 50 or 100 nM) during 24 h (incubation period).

### Analysis of cellular viability

To describe the cellular death induced by URB597, OEA or PEA, cultures were analyzed 24 h after drug treatments. Cellular viability was performed by methyl thiazolyl tetrazolium (MTT) assay [23,24] which evaluates the metabolic reduction of MTT active neurons quantified by the measuring of the formation of a dark blue formazan product. Briefly, cerebella neurons were plated in Petri multidishes with BME 10% fetal bovine serum and 1% penicillin/streptomycin. The neurons were serum deprived overnight and then stimulated with the respective treatments at 24 h. To study how the drugs affect the cellular viability, cells were incubated with MTT (40 μg/mL) for 15 min at 37°C and after medium removal, formed formazan blue was extracted with DMSO and quantified spectrophotometrically at 570 nm as described [25,26]. Under bright field, a photomicrograph was taken by one person blind to the experiment, and the cellular death index was calculated by the ratio of the number of dead neurons to the total number of cells in each field. Additionally, swollen soma and fragmented extensions were considered as a parameter to determinate cellular death. The final calculation was pooled from the data produced from four experiments in triplicate. Finally, to avoid experimental bias, at the end of the studies the code was broken to reveal the treatments of each MTT test.

### Statistical analysis

The data were expressed as mean ± S.E.M. The significance of differences between groups was evaluated by one-way analysis of variance (ANOVA) followed by a Scheffé-Test for multiple comparisons. Analyses were done with Statview Software (version 5.0.1; SAS Institute, Cary, NC. USA) and differences were considered significant if *p *< 0.05.

## Results

### The effects of URB597 on cellular death

**Figure 1:**
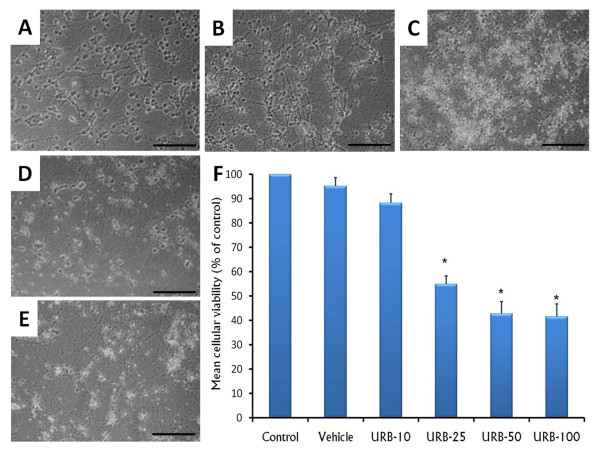
**Photomicrograph of cerebellar granule cells incubated only with culture media (control) or treated with URB597 at 10, 25, 50 or 100 nM (Panels A-E, respectively)**. The cellular viability (Panel F) was determined by MTT and data is presented as mean ± SEM (%). Scale bar, 100 μm (* vs control/vehicle, *p *< 0.05).

Since no differences were observed between control and vehicle groups only the photomicrograph of control group was included in the results. After 24 h of incubation, control group (Figure 1A) showed a confluent layer of cells with bright-phase cell bodies and spreading extensions. Upon exposure to different concentrations of URB597 (Figure 1B [10 nM], C [25 nM], D [50 nM], E [100 nM]), a decrease in cell viability in the MTT assay was observed. Importantly, the remaining cells revealed swollen soma and fragmented extensions. To determine if URB597 was diminishing the number of cerebellar granule neurons, we counted the cells after the pharmacological challenge. Statistical analysis showed significant effects were found in URB597-treated groups (ANOVA; *F*_(5,54)_= 3,69, *p *< 0.0001). Post-hoc analysis showed that URB597 (25, 50 or 100 nM) produced a significant decrease in the number of cerebellar granule neurons (Scheffé-Test: Control/Vehicle vs. URB-25 (25 nM), *p *< 0.0001; Control/Vehicle vs. URB-50 (50 nM), *p *< 0.0001; Control/Vehicle vs. URB-100 (100 nM), *p *< 0.0001; Figure 1F). We observed 50% cell death diminution after 24 h of incubation with URB597 (at the highest dose). This result is consistent with previous observations reported by others [27].

### The effects of OEA on cellular death

**Figure 2:**
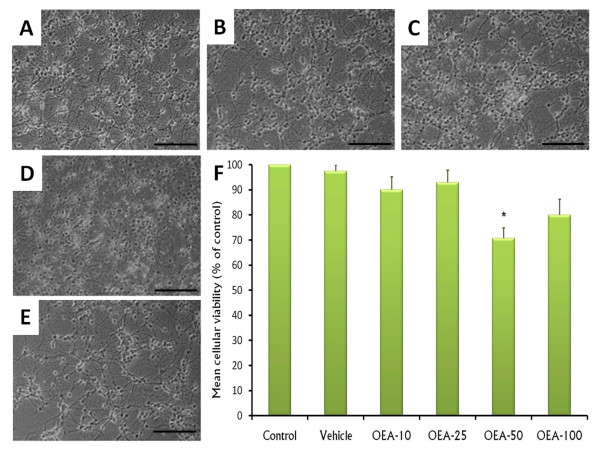
**Photomicrograph of cerebellar granule cells incubated only with culture media (control) or treated with OEA at 10, 25, 50 or 100 nM (Panels A-E, respectively), the remaining cells revealed swollen soma and fragmented extensions**. The cellular viability (Panel F) was determined by MTT and data is presented as mean ± SEM (%). Scale bar, 100 μm (* vs control/vehicle, *p *< 0.05).

Next, we analyzed the cellular viability in cerebellar granule neurons cultured during 24 h with different concentrations of OEA (10, 25, 50 or 100 nM). As shown in photomicrograph, control group (Figure 2A) showed that neurons had long processes with a bipolar morphology and round shape. In contrast, a diminution in neurites as well as swollen soma and fragmented extensions were observed after the treatment of OEA (Figure 2B [10 nM], C [25 nM], D [50 nM], E [100 nM]). To determine the number of cerebellar granule neurons after the treatments, we performed a count of the cells after the pharmacological challenge. We found that incubation of OEA (50 nM) decreased significantly the number of cerebellar granule neurons (ANOVA; *F*_(5,54)_= 5, 88, *p *< 0.0001; post-hoc analysis, Scheffé-Test: Control/Vehicle vs. OEA-50 (50 nM), *p *< 0.0001; Figure 2F). After 24 h, more that 20% of cerebellar granule neurons underwent cell death with the treatment of OEA (50 nM). It is worthy to mention that we found a resistance to cell death if cultured with OEA at 100 nM.

### The effects of PEA on cellular death

**Figure 3:**
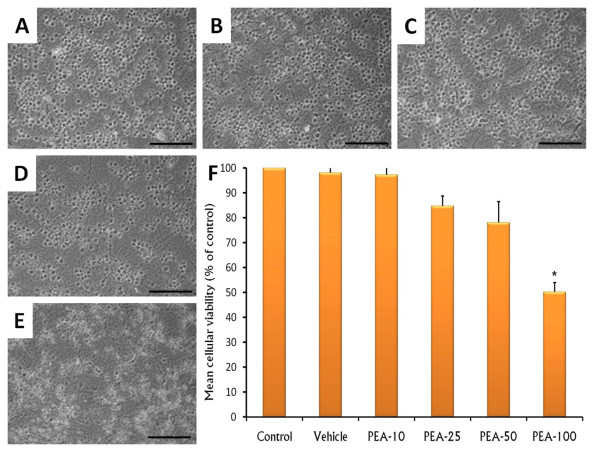
**Photomicrograph of cerebellar granule cells incubated only with culture media (control) or treated with PEA at 10, 25, 50 or 100 nM (Panels A-E, respectively)**. The cellular viability (Panel F) was determined by MTT and data is presented as mean ± SEM (%). Scale bar, 100 μm (* vs control/vehicle, *p *< 0.05).

To investigate whether PEA would induce cellular death, we analyzed cellular viability in cerebellar granule neurons after the treatment of PEA at different concentrations (10, 25, 50 or 100 nM). It was found that neurons in the control group (Figure 3A) were densely packed with healthy morphology whereas neurons incubated with PEA showed a diminution in cellular viability in the MTT assay. As shown in microphotography, PEA induced swollen soma and fragmented extensions (Figure 3B [10 nM], C [25 nM], D [50 nM], E [100 nM]). Next, it was determined the number of cerebellar granule neurons after the incubation with PEA. Statistical diminutions were found in the number of cerebellar granule neurons at the highest dose used of PEA (100 nM; ANOVA; *F*_(5,54)_= 9, 42, *p *< 0.0001, post-hoc analysis, Scheffé-Test: Control/Vehicle vs. PEA-100 (100 nM), *p *< 0.0001; Figure 3F).

## Discussion

The present study shows that inhibition of the FAAH activity using URB597 induces cellular death. Although the molecular mechanism underlying the observed results remain unknown, we can hypothesize from this study two mechanisms: Cellular death promoted by URB597 could be related with the endogenous accumulation of ANA as described by others [28,29]. In this regard, Fegley and colleagues reported that the administration of URB597 increases the endogenous levels of ANA [30], and it has been suggested that this endocannabinoid promotes cellular death as reported previously [20,22,31-34]. Nevertheless, the results in our study using URB597 confirm similar findings. For example, Siegmund and colleagues showed that hepatocytes pretreated with URB597 displayed an enhancement in ANA-induced reactive oxygen species formation and they were susceptible to ANA-mediated death [27].

The second route of action that may be linked in the effects observed in our report is related to the MAP Kinase activity. The cellular death caused by URB597 may involve the activation of this intracellular cascade, suggested as an important key element in apoptotic mechanisms [20-22]. Experimental evidence suggest that MAP Kinase is activated by endocannabinoids [35]. Further experiments aimed to describe the effects of URB597 on activity of MAP Kinase should be addressed.

We also found that OEA diminished neuronal survival. The present results are consistent with previous reports. For example, Ambrosini and colleagues reported that this lipid (at 2.5 nM) significantly reduces in vitro DNA strand breaks both in fertile and infertile subjects [36]. Since OEA is able to activate Ras-Erk cascade [37], one might think that this pathway may participate in the molecular mechanism of OEA to induce cellular death. It is known that Raf-1 and MEK/ERK are components of the Ras/ERK-dependent signal transduction cascade regulating cellular apoptosis [38,39]. However, the neurobiological role of Ras/ERK signal under the influence of OEA should be determined to fully understand the effects described in this report.

The final compound examined, PEA, showed a significant diminution in the number of cerebellar granule neurons (only at the highest dose). In agreement with this observation, Franklin *et al*. (2003) showed that PEA (at a dose of 100 μM) increased cellular death [40]. These results suggest that PEA might be modulating cellular viability. Despite that it is unknown the neurobiological mechanism activated by PEA to induce cellular death, Di Marzo *et al*. (2001) have proposed that PEA may act in synergy with ANA to potentiate the effects induced by this endocannabinoid [41]. In this regard, it has been described that PEA enhances the anti-proliferative effects of ANA on human breast cancer cells by inhibiting the expression of FAAH.

Although we did not describe in the current report a mechanism of action of URB597, OEA or PEA on cellular death, further studies aimed to test the role of the endocannabinoid system should be addressed. It would be worthy to test whether SR141716A, a selective CB_1 _cannabinoid receptor antagonist, is able to block the effects caused by URB597, OEA or PEA in cellular viability.

In conclusion, our studies describe that URB597, OEA or PEA induce cellular death in cerebellar granule neurons. The present results enhance the investigation about the neurobiological properties of these compounds on apoptosis.

## Competing interests

The authors declare that they have no competing interests.

## Authors' contributions

Conceived and designed the experiments: EMR, OAC. Performed the experiments: KL, GD, OAC, MPR, DMA, EMR. Analyzed the data and statistics: EMR, OAC. Contributed reagents/materials/analysis tools: JM, RDC. Wrote the paper: EMR. All authors were equally involved in reading and approving the final manuscript.
